# Effect Size in Surgical Intervention Into Shoulder: What Procedures Are Game Changers and What Are Not?

**DOI:** 10.5435/JAAOSGlobal-D-20-00022

**Published:** 2020-03-17

**Authors:** Ragu Paraparan, Patrick H. Lam, George A. C. Murrell

**Affiliations:** From the Orthopaedic Research Institute, St George Hospital Campus, University of New South Wales, Sydney, Kogarah, NSW, Australia.

## Abstract

A number of surgical procedures are performed to treat a variety of shoulder pathologies. What is currently not understood is which of these surgical interventions provide the most improvement in patient-reported pain and function.

**Questions:**

We aimed to determine, from a patient's perspective, which were the most effective commonly performed surgical procedures for disorders of the shoulder and which were not.

**Methods:**

This study was a retrospective analysis of prospectively collected data from patients who underwent shoulder surgery by a single surgeon. To be included, at least 20 patients needed to have undergone that procedure and completed a questionnaire evaluating their shoulders function preoperatively and 6 months postoperatively. The primary outcome was change in response to the question “how is your shoulder overall?” Effect size is reported as Cohen's *d* (standardized mean difference).

**Results:**

Two thousand two hundred six surgical procedures in 13 categories met the inclusion criteria. All procedures were associated with improvements in the patient-ranked overall shoulder status at 6 months (*P* < 0.01 to *P* < 0.0001). Reverse total shoulder arthroplasty (RTSA) provided the greatest effect size (improvement) in the overall shoulder status (d = 3.14, 95% CI, 2.49 to 3.79), followed by total shoulder arthroplasty (d = 2.60, 95% CI, 2.10 to 3.10) and capsular release (d = 1.41, 95% CI, 1.08 to 1.75). RTSA provided the greatest effect size in patient-reported shoulder pain, whereas capsular release provided the greatest effect size in patient-reported shoulder function. Acromioclavicular joint resection (d = 1.22, 95% CI, 0.56 to 1.88) and acromioplasty (d = 1.29, 95% CI, 0.96 to 1.61) provided the least effect size overall.

**Conclusion:**

All shoulder surgical procedures in this study provided a notable patient-perceived therapeutic benefit in a relatively short period of time (6 months). RTSA, total shoulder arthroplasty, and capsular release are the most effective procedures. Acromioplasty and acromioclavicular resection are the least effective.

A number of surgical procedures are performed to treat a variety of shoulder pathologies. What is currently not understood is which of these surgical interventions provide the most improvement in patient-reported pain and function.

Most studies assessing the outcomes of surgical procedures have used global scores with various weighting for shoulder pain, strength, function, and daily activity.^1,2,3,4^

Many studies have attempted to incorporate the patient perspective by asking about patient satisfaction with the procedure, with the patient responding if they were satisfied or not or if the shoulder got better or worse. However, these dichotomous responses provide no indication of the amount of improvement gained from the procedure.^5^

The aim of this study was to determine, from a patient's perspective, which are the most effective commonly performed surgical procedures for disorders of the shoulder, and which are not.

## Methods

### Study Design

This study was a retrospective analysis of prospectively collected data from patients who underwent shoulder surgery by one surgeon at a single campus between 2004 and 2015 to determine the effect size of different surgical interventions of the shoulder from a patient perspective. The primary outcome for this study was the change in Likert scale response to the question “how is your shoulder overall?” preoperatively to postoperatively at 6 months. Secondary outcomes were patient-perceived improvements in the frequency of pain, level of pain, stiffness, and function. To be included within the study, patients needed to have undergone primary shoulder surgery by a single shoulder surgeon (G.A.C.M.). For a procedure to be included in the analysis, a minimum of 20 patients were required to have undergone that procedure. Patients having two surgical procedures at the same time were also included, provided that at least 20 patents had undergone that combination of procedures. Patients were excluded from the study if they had a concomitant shoulder fracture, underwent revision surgery, or did not attend the 6-month follow-up.

### Outcome Measures

Patients received a modified L'Insalata questionnaire^6^ before surgery and 6 months after surgery. Patients were asked within the 14-question L'Insalata questionnaire to rank their overall shoulder status, frequency of pain, level of pain, and functional level using the Likert scales. The responses were converted to ordinal numerical values for statistical analysis. For example, for our primary outcome question “how is your shoulder overall,” there were five possible responses on a Likert scale graded from “very bad,” “bad,” “poor,” “fair,” and “good” which were assigned the numbers 0, 1, 2, 3, 4, respectively. The secondary outcomes for this study was patient-reported frequency of pain graded from “daily” to “none,” the level of pain graded from “very severe” to “none,” and difficulty with activities graded from “very severe” to “none.” A full version of the L'Insalata questionnaire is attached as an appendix (See additional material A, http://links.lww.com/JG9/A69).

### Surgical Technique

All surgeries analyzed within this study were performed under interscalene block by a single surgeon with the patient placed within the beach chair position. Surgical intervention into the shoulder was either undertaken arthroscopically or open. Acromioclavicular joint resection for acromioclavicular joint arthritis, acromioplasty for rotator cuff impingement, Bankart repair and superior labral anterior to posterior (SLAP) repair for labral tears, calcific débridement for calcific tendinitis, capsular release for idiopathic adhesive capsulitis, polytetrafluoroethylene (PTFE) patch rotator cuff repair, and rotator cuff repair for rotator cuff tears were performed using an arthroscopic technique. Open shoulder surgery was undertaken for the shoulder arthroplasties, specifically anatomic total shoulder arthroplasty (TSA), reverse total shoulder arthroplasty (RTSA), and hemiarthroplasty. Hemiarthroplasty was performed for cuff insufficient patients with arthritis (before the advent of reverse total shoulders) and for young patients with arthritis. The indication for TSA was severe glenohumeral arthritis, for RTSA was cuff tear arthropathy, and hemiarthroplasty was indicated for both glenohumeral arthritis and cuff tear arthropathy.

### Statistical Analysis

A Wilcoxon signed-rank test was used to evaluate the significance of 6 month postoperative outcomes within each surgical intervention group. The Kruskal-Wallis test with Dunn's correction was used to determine whether notable differences in shoulder improvement at 6 months postoperatively existed between surgical groups. **E**ffect size was calculated as the standardized mean difference. The standardized mean difference was calculated by taking the mean difference in preoperative and postoperative response on the Likert scale and dividing this by the pooled preoperative and postoperative SD to give a standardized mean difference as Cohen's *d* for each outcome.

## Results

### Study Group

Between 2004 and 2015, 3,201 surgeries had been undertaken by one surgeon. Of the 3,201 patients, 64 did not meet the inclusion criteria because they had a surgical procedure that was performed in less than the minimum requirement of 20 procedures. Two hundred fifty-seven patients who underwent revision surgery, 24 patients treated for shoulder fractures, 650 patients did not return for follow-up at 6 months or failed to complete any aspect of the questionnaire preoperatively 6 months were excluded, leaving 2,206 patients for the study.

Of these 2,206 patients, 1,577 underwent rotator cuff repair, 87 arthroscopic acromioplasty, 86 capsular release, 84 Bankart repair, 74 SLAP repair, 56 TSA, 53 PTFE patch rotator cuff repair, 43 rotator cuff repair with capsular release, 41 RTSA, 37 rotator cuff repair with stabilization, 25 calcific tendinitis débridement, 22 hemiarthroplasty, and 21 acromioclavicular joint resection. The demographics of each group are shown in Table 1).

**Table 1:** Demographics for Each Surgical Intervention of the Shoulder

Type of Surgery	No. of Patients	Male:Female	Age	Surgical Time (min)	Public:Private	Workers Compensation Cases (% of Total Cases)
Rotator cuff repair	1,577	869:708	59 ± 0.3 (15-91)	22 ± 0.3 (4-110)	140:1,437	430 (27)
Acromioplasty	87	45:42	45 ± 1.3 (19-75)	23.1 ± 0.9 (10-50)	5:82	35 (40)
Capsular release	86	35:51	55 ± 0.6 (40-68)	24.6 ± 1.2 (8-60)	2:84	31 (36)
Bankart repair	84	64:20	28 ± 1.1 (12-60)	29.5 ± 1.3 (6-65)	4:80	13 (15)
SLAP repair	74	63:11	36 ± 1.2 (19-56)	28.6 ± 1.6 (6-70)	4:70	25 (33)
Total shoulder arthroplasty	56	32:24	68 ± 1.6 (48-90)	105.9 ± 3.1 (60-200)	2:54	1 (2)
PTFE patch rotator cuff repair	53	38:15	66 ± 1.4 (46-88)	48.3 ± 2.7 (6-117)	4:49	16 (30)
Rotator cuff repair + capsular release	43	16:27	57 ± 1.4 (40-72)	27.1 ± 2.1 (5-60)	3:40	14 (33)
Reverse total shoulder arthroplasty	41	9:32	76 ± 1.3 (61-91)	95.4 ± 4.6 (14-160)	7:34	3 (7)
Rotator cuff repair + stabilization	37	29:8	44 ± 1.8 (21-71)	29.9 ± 2.2 (12-65)	0:37	15 (40)
Calcific débridement	25	8:17	51 ± 2.2 (31-70)	26.6 ± 1.6 (13-45)	1:24	2 (8)
Hemiarthroplasty	22	7:15	71 ± 4.2 (51-85)	75.2 ± 5.7 (40-120)	0:22	0 (0)
Acromioclavicular joint resection	21	14:7	39 ± 3.7 (17-57)	44.1 ± 4.5 (25-90)	9:12	12 (57)

PTFE = polytetrafluoroethylene, SLAP = superior labral anterior to posterior, SEM = standard error of measurement

±SEM, (minimum value to maximum value).

### Overall Shoulder Status

The primary outcome for this study was the change in the patient-ranked overall shoulder status. All shoulder surgery groups had a statistically significant improvement in the overall shoulder status at 6 months postoperatively (*P* < 0.01 to *P* < 0.0001).

RTSA (d = 3.14, 95% CI, 2.49 to 3.79) provided the greatest effect size in the overall shoulder status, followed by TSA (d = 2.60, 95% CI, 2.10 to 3.10) and capsular release surgery 2.29 (95% CI, 1.85 to 2.61). The poorest performing shoulder surgeries in the patient-assessed overall shoulder status at 6 months were acromioclavicular joint resection and acromioplasty which had the effect sizes of 1.22 (95% CI, 0.56 to 1.88) and 1.29 (95% CI, 0.96 to 1.61), respectively (Figuress 1 and 2).

**Figure 1:**
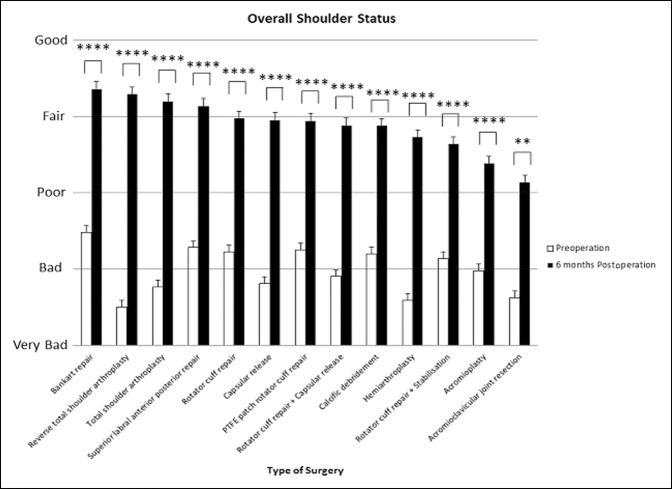
Chart showing mean (±standard error of measurement) preoperative and 6-month postoperative patient-reported overall shoulder status. ****P < 0.0001, ***P < 0.0001, **P < 0.01, and *P < 0.05 (using Wilcoxon signed-rank test). PTFE = polytetrafluoroethylene

**Figure 2:**
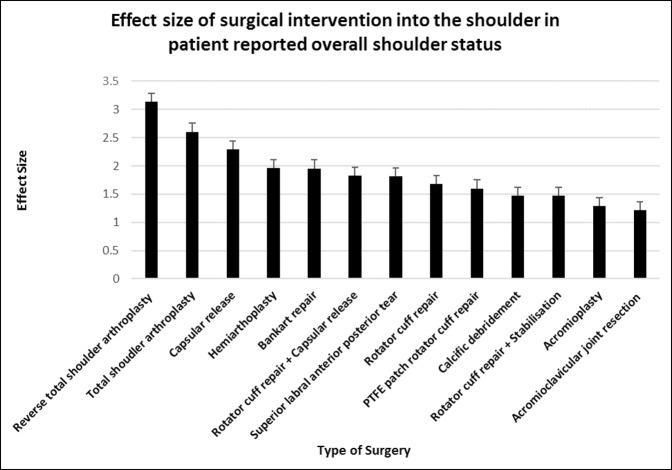
Chart showing mean (±standard error of measurement) effect size of surgical intervention into the shoulder in patient-reported overall shoulder status at 6 months postoperatively. PTFE = polytetrafluoroethylene

Reverse total shoulder arthroplasty provided a significantly greater improvement in the patient-reported overall shoulder status than Bankart repair, SLAP repair, rotator cuff repair, PTFE patch repair, calcific débridement, rotator cuff repair + stabilization, and acromioplasty (*P* < 0.05 to *P* < 0.0001). TSA provided a significantly greater improvement in the overall shoulder status than rotator cuff repair, rotator cuff + stabilization, and acromioplasty (*P* < 0.01 to *P* < 0.0001). Capsular release had significantly greater improvement in the patient-reported overall shoulder status than acromioplasty (*P* < 0.05).

### Level of Shoulder Pain at Rest

All surgeries of the shoulder provided a notable reduction in the level of pain at rest at 6 months (*P* < 0.05 to *P* < 0.0001).

**Figure 3:**
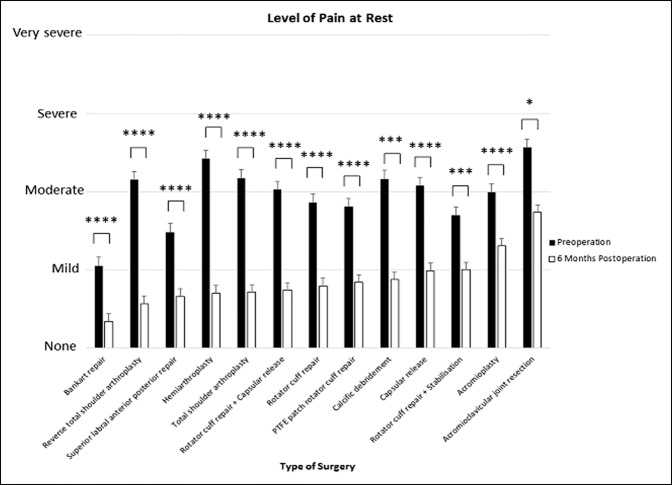
Chart showing mean (±standard error of measurement) preoperative and 6-month postoperative patient-reported level of shoulder pain at rest. ****P < 0.0001, ***P < 0.001, and *P < 0.05 (using Wilcoxon signed-rank test). PTFE = polytetrafluoroethylene

**Figure 4:**
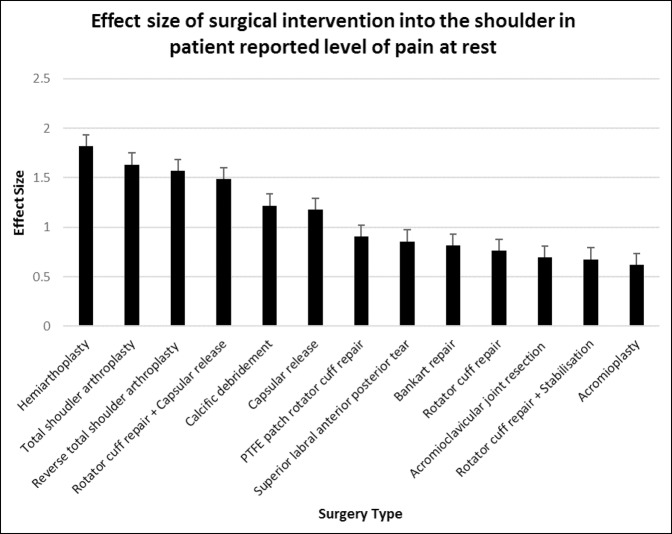
Chart showing mean (±standard error of measurement) effect size of surgical intervention into the shoulder in patient-reported level of pain at rest at 6 months postoperatively. PTFE = polytetrafluoroethylene

The shoulder arthoplasties provided the most improvement in the level of shoulder pain at rest, with hemiarthroplasty (d = 1.82, 95% CI, 1.12 to 2.52) giving the most improvement, followed by TSA (d = 1.63, 95% CI, 1.21 to 2.06) and then RTSA (d = 1.57, 95% CI, 1.08 to 2.07). The shoulder arthoplasties were associated with improvements in patient-ranked pain at rest from “moderate/severe” to “none/mild” after 6 months (*P* < 0.0001). Acromioplasty (d = 0.62, 95% CI, 0.31 to 0.92) had the least effect, followed by rotator cuff repair + stabilization (d = 0.68, 95% CI, 0.21 to 1.15), and acromioclavicular joint resection (d = 0.70, 95% CI, 0.08 to 1.32) (Figures 3 and 4).

### Level of Shoulder Pain With Overhead Activities

All surgical interventions into the shoulder except for acromioclavicular joint resection resulted in a significant reduction in the level of shoulder pain experienced with overhead activities (*P* < 0.01 to *P* < 0.0001).

**Figure 5:**
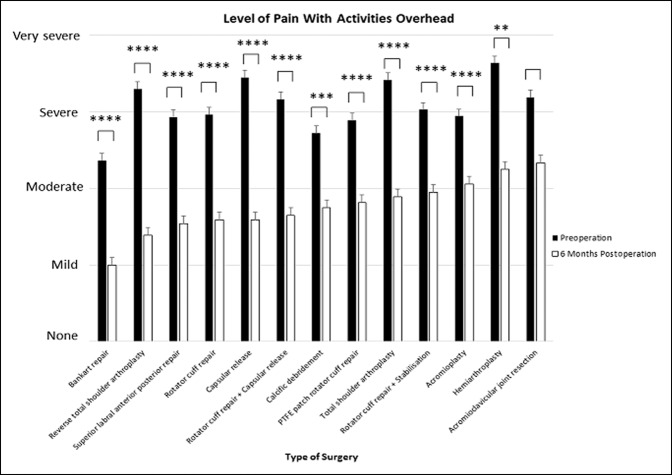
Chart showing mean (±standard error of measurement) preoperative and 6-month postoperative patient-reported level of pain with overhead activity. ****P < 0.0001, ***P < 0.0001, **P < 0.01, and *P < 0.05 (using Wilcoxon signed-rank test). PTFE = polytetrafluoroethylene

**Figure 6:**
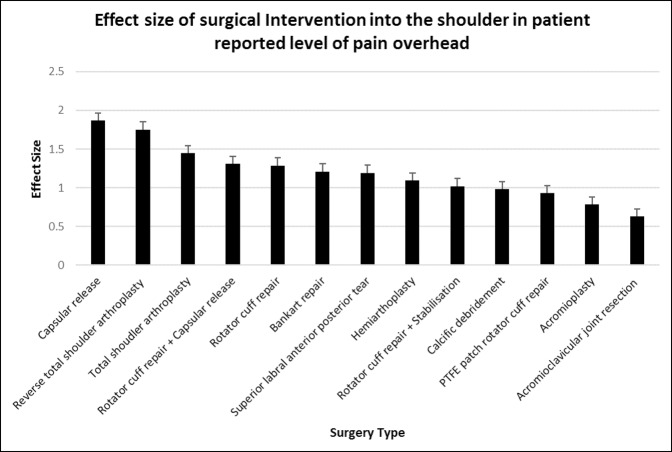
Chart showing mean (±standard error of measurement) effect size of surgical intervention into the shoulder in patient-reported level of pain overhead at 6 months postoperatively. PTFE = polytetrafluoroethylene

The greatest improvement in the patient-reported level of overhead of pain was provided by capsular release (d = 1.87, 95% CI, 1.51 to 2.23) and then RTSA (d = 1.75, 95% CI, 1.24 to 2.26) in which patients reported that the level of pain experienced improved from “severe/very severe” to “mild/moderate” (*P* < 0.0001). Acromioclavicular joint resection provided no statistically significant improvement. Acromioplasty (d = 0.78, 95% CI, 0.47 to 1.09) also had little effect on pain during overhead activity, with patients reporting a mean improvement of roughly “severe” pain preoperatively to “moderate” pain postoperatively at 6 months (*P* < 0.0001) (Figures 5 and 6).

### Frequency of Pain With Activity

All surgeries of the shoulder except for acromioclavicular joint resection significantly reduced the frequency of pain with activity (*P* < 0.001 to *P* < 0.0001).

**Figure 7:**
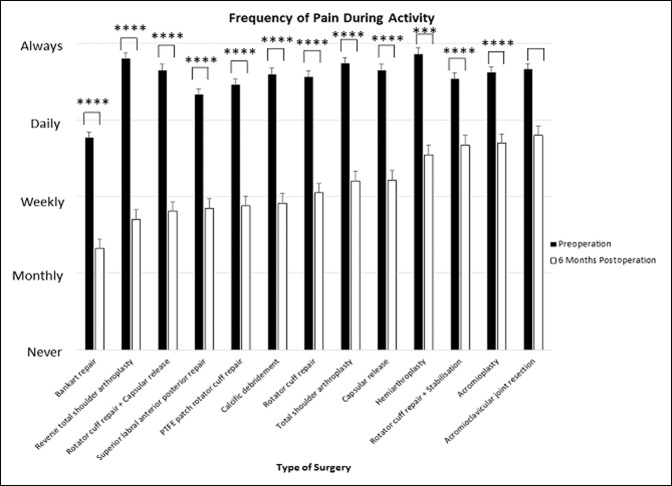
Chart showing mean (±standard error of measurement) preoperative and 6-month postoperative patient-reported frequency of pain during activity. ****P < 0.0001, ***P < 0.0001, **P < 0.01, and *P < 0.05 (using Wilcoxon signed-rank test). PTFE = polytetrafluoroethylene

**Figure 8:**
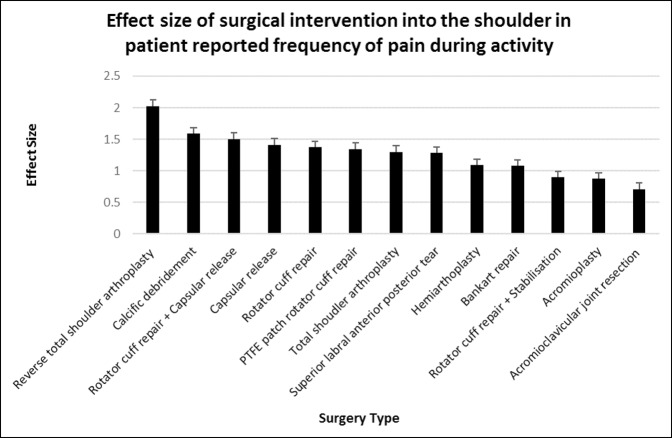
Chart showing mean (±standard error of measurement) effect size of surgical intervention into the shoulder in patient-reported frequency of pain during activity at 6 months postoperatively. PTFE = polytetrafluoroethylene

RTSA (d = 2.03, 95% CI, 1.51 to 2.55) reported the most improvement in frequency of pain during activity, followed by calcific débridement (d = 1.59, 95% CI, 0.95 to 2.23), rotator cuff repair + capsular release (d = 1.50, 95% CI, 1.03 to 1.98) and then capsular release (d = 1.41, 95% CI, 1.08 to 1.75). For RTSA patients, their frequency of pain with activity improved from “always” to less than “weekly.” The frequency of pain during activity for calcific débridement, rotator cuff repair + capsular release, and capsular release patients reduced from “always/daily” to closer to “weekly.” Acromioclavicular joint resection provided no improvement in the frequency of pain during activity. Acromioplasty (d = 0.872, 95% CI, 0.56 to 1.18) and rotator cuff repair + stabilization (d = 0.895, 95% CI, 0.42 to 1.37) provided relatively small effect sizes, with patients improving from “always/daily” to closer to “daily” frequency of pain with activity (Figures 7 and 8).

### Difficulty With Overhead Activities

Acromioclavicular joint resection and rotator cuff repair + stabilization provided no notable improvement in difficulty patients experienced with overhead activities. All other shoulder surgeries resulted in a significant reduction in patient-perceived difficulty with overhead activities (*P* < 0.01 to *P* < 0.0001).

**Figure 9:**
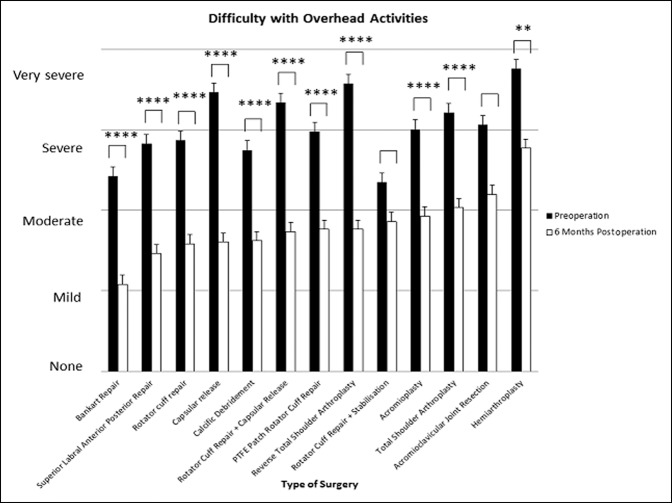
Chart showing mean (±standard error of measurement) preoperative and 6-month postoperative patient-reported difficulty with overhead activities. ****P < 0.0001, ***P < 0.0001, **P < 0.01, and *P < 0.05 (using Wilcoxon signed-rank test). PTFE = polytetrafluoroethylene

**Figure 10:**
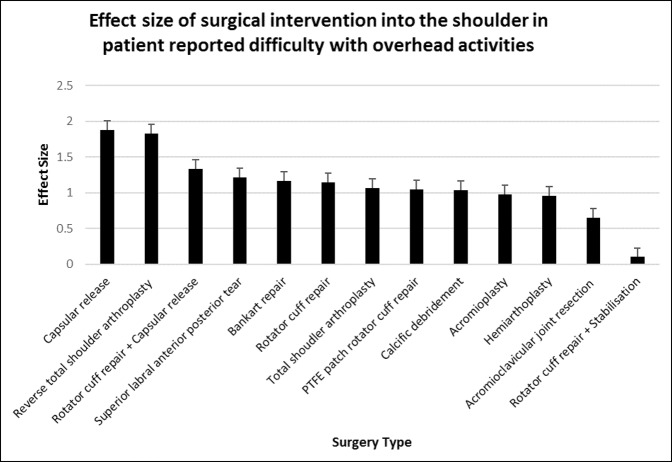
Chart showing effect size of surgical intervention into the shoulder in patient-reported difficulty with overhead activities at 6 months postoperatively. PTFE = polytetrafluoroethylene

Capsular release (d = 1.88, 95% CI, 1.52 to 2.24) provided the greatest effect size in improving patient-reported difficulty with overhead activity, followed by RTSA (d = 1.83, 95% CI 1.39 to 2.27) and rotator cuff repair + capsular release. Patients undergoing these surgeries experienced “severe/very severe” difficulty with overhead activities and improved to “mild/moderate” difficulty postoperatively at 6 months (*P* < 0.0001). Acromioplasty had a limited effect size of 0.98 (95% CI, 0.66 to 1.29), with patients preoperatively reporting “severe” difficulty with overhead activities and closer to “moderate” postoperatively (*P* < 0.0001). Hemiarthroplasty (d = 0.96, 95% CI, 0.33 to 1.58) also provided a poor effect size, with patients improving from “very severe” to “severe” difficulty with overhead shoulder activity (*P* < 0.01) (Figures 9 and 10).

## Discussion

The hypothesis for this study was that arthroscopic capsular release for idiopathic adhesive capsulitis would provide the largest effect size in patient-reported overall shoulder status based on the limited findings within the literature.^7-9^ Our findings show that RTSA, followed by TSA and then capsular release provided the most benefit in patient-perceived overall shoulder status at 6 months postoperation. RTSA provided the greatest effect size in patient-reported shoulder pain. Capsular release provided the greatest effect size of all shoulder surgeries in patient-rated function.

In this study, all three types of shoulder arthoplasties provided excellent pain relief at rest. The three versions of shoulder arthoplasties ranked in the top four of all shoulder surgical interventions for effect size in the patient-reported level of pain at rest. RTSA resulted in the most benefit for pain outcomes when the shoulder was in motion, whereas TSA and hemiarthroplasty provided less pain relief during shoulder motion. In our study, RTSA provided the most improvement in patient-reported frequency of pain with activity and the second best improvement in pain level, with overhead activity of all 13 shoulder surgeries in this study. In comparison, TSA and hemiarthroplasty ranked seventh and ninth for patient-reported improvement in the frequency of pain with activity, respectively. For pain relief with overhead activities, TSA ranked third and hemiarthroplasty eighth.

More evident differences between the shoulder arthoplasties were observed in functional outcomes. RTSA provided the second greatest effect size in overhead shoulder function. TSA resulted in a relatively modest improvement in shoulder function, ranking for seventh overhead function. Hemiarthroplasty provided a relatively poor effect size for patient-reported overhead function, ranking 11th.

Our findings support the evidence that RTSA and TSA are both superior in pain and functional outcomes to hemiarthroplasty.^10-12^ The findings of this study suggest that RTSA provides superior pain relief when the shoulder is in motion compared with both TSA and hemiarthroplasty.

### Which Operations Were Not Game Changers?

Acromioplasty and acromioclavicular joint resection provided the poorest patient-reported improvement in overall shoulder status, with these patients rating their shoulders as “poor” postoperatively at 6 months.

Acromioplasty was one of the most commonly performed surgeries in the Western world, with a reported incidence of 101.9 per 100,000 in New York alone.^13^ Recently, several systematic reviews and one randomized clinical trial have begun to question the effectiveness of acromioplasty as a surgical procedure.^14-16^ Acromioplasty placed in the bottom four of all shoulder surgeries for both effect size and postoperative result in all outcomes measured in this study, except for difficulty with behind back activities. Acromioplasty was especially poor for pain outcomes when the shoulder was not in motion. Acromioplasty provided the least effect size and the second worst postoperative result in the level of pain at rest.

Acromioclavicular joint resection ranked in the bottom three of all surgical interventions for all measured outcomes. The limited literature on the outcome of acromioclavicular joint resection also shows minimal benefit associated with the procedure.^17,18,19,20^ We hypothesize that simply removing the distal portion of the clavicle for arthritis of the acromioclavicular joint does not adequately address the underlying pathology.

### Strengths and Limitations of the Study

There are a number of strengths to this study. All surgical interventions into the shoulder were completed by one surgeon at one center with all patients completing the same questionnaire, and hence, this study represents a highly valid comparison of all common shoulder surgeries. The L'Insalata questionnaire is a validated questionnaire that can dissociate between specific pain and functional outcomes.^6^ Finally, most shoulder surgeries under comparison in this study had equivalent or larger patient numbers to comparable studies.

However, there are several limitations to the findings of this study. This study was a retrospective cohort study. The high internal validity of this study may limit the applicability of these findings to other settings. The follow-up period for this study was 6 months, which is shorter than most studies evaluating shoulder surgery outcome. Finally, it is important to consider that different surgical interventions were often for different indications.

## Conclusion

All shoulder surgical procedures within this study provided a notable therapeutic effect size in a relatively short period of time (6 months). However, some procedures provided a greater improvement in patient-reported outcomes than others. Shoulder replacement, especially RTSA, provided the greatest improvement in the patient-reported shoulder status. We hypothesized that capsular release for idiopathic adhesive capsulitis would provide the greatest effect size in patient-assessed overall shoulder status; however, it ranked third best of all shoulder surgeries. Capsular release however provided the greatest effect size in patient-rated overhead function. Conversely, acromioplasty and acromioclavicular joint resections were associated with the least benefit in patient-assessed shoulder pain and function, suggesting that in these procedures the underlying pathological process may have not been appropriately addressed.

## References

[1] Amstutz HC Sew Hoy AL Clarke IC: UCLA anatomic total shoulder arthroplasty. Clin Orthop Relat Res 1981;7-20. 7226634

[2] Constant CR Gerber C Emery RJ Sojbjerg JO Gohlke F Boileau P: A review of the constant score: Modifications and guidelines for its use. J Shoulder Elbow Surg 2008;17:355-361. 18218327 10.1016/j.jse.2007.06.022

[3] Harvie P Pollard TC Chennagiri RJ Carr AJ: The use of outcome scores in surgery of the shoulder. J Bone Joint Surg Br 2005;87:151-154. 15736732 10.1302/0301-620x.87b2.15305

[4] Michener LA McClure PW Sennett BJ: American shoulder and Elbow surgeons standardized shoulder assessment form, patient self-report section: Reliability, validity, and responsiveness. J Shoulder Elbow Surg 2002;11:587-594. 12469084 10.1067/mse.2002.127096

[5] Kirkley A Griffin S Dainty K: Scoring systems for the functional assessment of the shoulder. Arthroscopy 2003;19:1109-1120. 14673454 10.1016/j.arthro.2003.10.030

[6] L'Insalata JC Warren RF Cohen SB Altchek DW Peterson MG: A self-administered questionnaire for assessment of symptoms and function of the shoulder. J Bone Joint Surg Am 1997;79:738-748. 9160947

[7] Baums MH Spahn G Nozaki M Steckel H Schultz W Klinger HM: Functional outcome and general health status in patients after arthroscopic release in adhesive capsulitis. Knee Surg Sports Traumatol Arthrosc 2007;15:687. 27520759 10.1007/s00167-007-0314-z

[8] Le Lievre HM Murrell GA: Long-term outcomes after arthroscopic capsular release for idiopathic adhesive capsulitis. J Bone Joint Surg Am 2012;94:1208-1216. 22760389 10.2106/JBJS.J.00952

[9] Nicholson GP: Arthroscopic capsular release for stiff shoulders: Effect of etiology on outcomes. Arthroscopy 2003;19:40-49. 12522401 10.1053/jars.2003.50010

[10] Leung B Horodyski M Struk AM Wright TW: Functional outcome of hemiarthroplasty compared with reverse total shoulder arthroplasty in the treatment of rotator cuff tear arthropathy. J Shoulder Elbow Surg 2012;21:319-323. 21872496 10.1016/j.jse.2011.05.023

[11] Radnay CS Setter KJ Chambers L Levine WN Bigliani LU Ahmad CS: Total shoulder replacement compared with humeral head replacement for the treatment of primary glenohumeral osteoarthritis: A systematic review. J Shoulder Elbow Surg 2007;16:396-402. 17582789 10.1016/j.jse.2006.10.017

[12] van den Bekerom MP Geervliet PC Somford MP van den Borne MP Boer R: Total shoulder arthroplasty versus hemiarthroplasty for glenohumeral arthritis: A systematic review of the literature at long-term follow-up. Int J Shoulder Surg 2013;7:110-115. 24167403 10.4103/0973-6042.118915 PMC3807945

[13] Shi LE Edwards TB: The role of acromioplasty for management of rotator cuff problems: Where is the evidence? Adv Orthopedics 2012;2012:5. 10.1155/2012/467571 PMC3535880 23316375

[14] Frank JM Chahal J Frank RM Cole BJ Verma NN Romeo AA: The role of acromioplasty for rotator cuff problems. Orthop Clin North Am 2014;45:219-224. 24684915 10.1016/j.ocl.2013.12.003

[15] Seitz AL McClure PW Finucane S Boardman ND III Michener LA: Mechanisms of rotator cuff tendinopathy: Intrinsic, extrinsic, or both? Clin Biomech (Bristol, Avon) 2011;26:1-12. 10.1016/j.clinbiomech.2010.08.001 20846766

[16] Singh C Patrick L Murrell G: Is acromioplasty of benefit for rotator cuff repair? Tech Shoulder Elbow Surg 2015;16:32-37.

[17] Bigliani LU Nicholson GP Flatow EL: Arthroscopic resection of the distal clavicle. Orthop Clin North Am 1993;24:133-141. 8421606

[18] Levine WN Barron OA Yamaguchi K Pollock RG Flatow EL Bigliani LU: Arthroscopic distal clavicle resection from a bursal approach. Arthroscopy 1998;14:52-56. 9486333 10.1016/s0749-8063(98)70120-3

[19] Novak PJ Bach BR Jr Romeo AA Hager CA: Surgical resection of the distal clavicle. J Shoulder Elbow Surg 1995;4:35-40. 7874563 10.1016/s1058-2746(10)80006-0

[20] Pensak M Grumet RC Slabaugh MA Bach BR Jr: Open versus arthroscopic distal clavicle resection. Arthroscopy 2010;26:697-704. 20434670 10.1016/j.arthro.2009.12.007

